# A novel Corchorus olitorius-derived biochar/Bi_12_O_17_Cl_2_ photocatalyst for decontamination of antibiotic wastewater containing tetracycline under natural visible light

**DOI:** 10.1038/s41598-023-38715-4

**Published:** 2023-08-14

**Authors:** Mahmoud Samy, Mohamed Gar Alalm, Ribh S. abodlal, Ali El-Dissouky, Mohamed N. Khalil, Ehab R. El-Helow, Tarek E. Khalil, Ahmed Tawfik

**Affiliations:** 1 Public Works Engineering Department, Faculty of Engineering, Mansoura University, Mansoura, 35516 Egypt; 2 Chemistry Department, Faculty of Science, Alexandria University, Alexandria, Egypt; 3 Water Pollution Research Department, National Research Centre, P.O. Box 12622, Giza, Egypt; 4 Department of Botany and Microbiology, Faculty of Science, Alexandria University, Alexandria, Egypt

**Keywords:** Environmental sciences, Materials science

## Abstract

Herein, a novel composite of Corchorus olitorius-derived biochar and Bi_12_O_17_Cl_2_ was fabricated and utilized for the degradation of tetracycline (TC) in a solar photo-oxidation reactor. The morphology, chemical composition, and interaction between the composite components were studied using various analyses. The biochar showed a TC removal of 52.7% and COD mineralization of 59.6% using 150 mg/L of the biochar at a pH of 4.7 ± 0.5, initial TC concentration of 163 mg/L, and initial COD of 1244 mg/L. The degradation efficiency of TC increased to 63% and the mineralization ratio to 64.7% using 150 mg/L of bare Bi_12_O_17_Cl_2_ at a pH of 4.7 ± 0.5, initial TC concentration of 178 mg/L, and COD of 1034 mg/L. In the case of biochar/Bi_12_O_17_Cl_2_ composite, the degradation efficiency of TC and COD mineralization ratio improved to 85.8% and 77.7% due to the potential of biochar to accept electrons which retarded the recombination of electrons and holes. The synthesized composite exhibited high stability over four succeeding cycles. According to the generated intermediates, TC could be degraded to caprylic acid and pentanedioic acid via the frequent attack by the reactive species. The prepared composite is a promising photocatalyst and can be applied in large-scale systems due to its high degradation and mineralization performance in a short time besides its low cost and stability.

## Introduction

The wide use of pharmaceuticals (e.g., antibiotics) for suppressing bacterial growth in humans and animals results in the uncontrolled release of antibiotics into the aquatic ecosystem^1,2^. Tetracycline (TC), an antibiotic, is continuously used in anti-infective treatment due to its low-cost and broad effectiveness towards various types of bacteria^3,4^. The increase of TC residues in aquatic life causes the development of tetracycline-resistant bacteria that can lead to the deterioration of human health^5,6^. Traditional treatment processes (e.g., biological treatment, adsorption, membrane filtration) cannot efficiently remove tetracycline due to its poor biodegradability and stability as well as the traditional technologies are expensive and produce secondary pollutants^7,8^. Thus, it is fateful to control the release of antibiotics (e.g., tetracycline) to water streams via the development of an efficient and low-cost treatment technology^9,10^.

Recently, advanced oxidation processes (AOPs) have exhibited excellent performance towards the degradation of refractory pollutants (e.g., antibiotics)^11,12^. Howbeit, some AOPs (e.g., Fenton, ozonation) are costly and generate secondary contaminants which obstruct the full-scale application^13–16^. Further, electrochemical AOPs cannot be applied on a larger scale due to the high cost and short service time of electrodes^17,18^. Photocatalysis process, one of the AOPs, is featured by its inexpensiveness, green and sustainable nature and effective degradation and mineralization performance towards bio-resistant pollutants via the generated reactive oxygen species (ROS) which qualify this technique to be implemented on a larger scale^19–21^. Nonetheless, the conventional photocatalysts such as TiO_2_ and ZnO have some flaws such as the wide bandgap and rapid reunite of charge carriers which inhibit the efficient degradation of bio-resistant pollutants and the utilization of large portion of solar intensity^22,23^. Therefore, it is imperative to design an efficient photocatalyst with a narrow bandgap and reduced recombination rate which contributes to the efficient degradation of pollutants and enhances the practical implementation of photocatalysis process.

Bismuth oxychloride (Bi_12_O_17_Cl_2_) photocatalyst has gained remarkable attention due to its ability to be utilized in photocatalysis process under visible light irradiation as well as its stability, excellent oxidation potential and non-toxicity^24,25^. Howbeit, the degradation performance by Bi_12_O_17_Cl_2_ is modest because of the inefficient separation of electrons and holes^26^. To overcome the aforementioned issue, researchers have fabricated Bi_12_O_17_Cl_2_-based heterojunction photocatalysts to suppress the concourse between charge carriers such as AgI/Bi_12_O_17_Cl_2_^27^, BiOBr/Bi_12_O_17_Cl_2_^28^ and Bi_12_O_17_Cl_2_/β-Bi_2_O_3_^29^. Howbeit, the preparation of these heterojunction photocatalysts increases the cost of treatment and the consumption of toxic chemicals.

Herein, dry stalks of Corchorus olitorius were compiled from the waste of food industry facility in Egypt and converted to a biochar via the pyrolysis in a muffle furnace under free-oxygen conditions followed by the preparation of a novel biochar/Bi_12_O_17_Cl_2_ heterojunction. The employment of a composite of Bi_12_O_17_Cl_2_ and carbonaceous material has not been extensively studied, as only a few studies reported the preparation of carbonaceous materials/Bi_12_O_17_Cl_2_ heterojunctions. Zhou et al. synthesized a composite of Bi_12_O_17_Cl_2_ and carbon-doped carbon nitride for the degradation of tetracycline and the degradation rate in the case of case of the composite was higher than that of bare Bi_12_O_17_Cl_2_ by 2.9 times^24^. The preparation of biochar/Bi_12_O_17_Cl_2_ composite can participate in improving the photodegradation performance due to the potential of biochar to act as an electron acceptor which inhibits the recombination between the charge carriers^30^. Further, introducing biochar can improve the adsorption capacity of the composite due to the expected high surface area and porosity of the biochar^31^. Additionally, the treatment cost can be reasonable in the case of using the biochar instead of the above-mentioned materials in Bi_12_O_17_Cl_2_-based heterojunction photocatalysts due to the availability of Corchorus olitorius produced from the food industry. On the other hand, converting Corchorus olitorius wastes to biochar contributes to solving the problems associated with their management and decreases the dependance on landfills. The disposal of wastes into landfills results in the contamination of soil, groundwater and air^32^. Thus, the utilization of biochar in photocatalysis process not only enhances the degradation performance, but also reduces the environmental hazards related to the improper management of solid waste.

In this study, a novel composite of Bi_12_O_17_Cl_2_ and Corchorus olitorius-derived biochar was fabricated and employed in photocatalysis process for the degradation of tetracycline. The degradation of tetracycline using Bi_12_O_17_Cl_2_, the synthesized biochar and biochar/Bi_12_O_17_Cl_2_ composite was carried out under different doses to determine the optimum doses. Further, the recyclability performance of the composite and the degradation mechanism were studied. Additionally, the generated intermediates were recognized, and the degradation pathways were suggested.

## Materials and methods

### Materials and chemicals

Sodium hydroxide (NaOH, 97%) was procured from Fisher chemical, Ltd. Absolut ethanol (C_2_H_6_O, 100%) and bismuth chloride (BiCl_3_, 97%) were obtained from Emplura, Ltd and Alpha Chemika, respectively. Fresh Corchorus olitorius stalks were harvested from the wastes of food industry facility in Giza, Egypt. Real industrial effluents were compiled from a pharmaceutical industrial facility in Giza, Egypt. The collected wastewater was left for 24 h to provide the time for suspended solids to settle, and then samples were decanted. The characteristics of the wastewater are provided in Table 1.

**Table 1:** Characteristics of real pharmaceutical wastewater.

Parameter	Min	Max	Average	STDEV
pH value	4.3	5.1	4.7	0.5
COD_t_ (mg/L)	1034	1244	1139	122.7
COD_s_ (mg/L)	934	988	961	67.8
COD_p_ (mg/L)	100	256	178	10.7
Tetracycline (mg/L)	163	178	170.5	15.7

### Preparation of Corchorus olitorius-derived biochar and biochar/Bi_12_O_17_Cl_2_ composite

Dry stalks of Corchorus olitorius were harvested from the wastes of food industry facility located in Giza, Egypt. The preparation of biochar from Corchorus olitorius started by drying the stalks at room temperature for 24 h, followed by grinding the stalks to a fine powder and washing with distilled water to remove any impurities. Subsequently, the powder was dried in an oven at 60 °C, followed by sieving using a 425 µm mesh sieve and pyrolyzing the powder for 2 h at 600 °C in a muffle furnace in the absence of oxygen. After cooling the biochar at room temperature, the prepared biochar was activated by adding 50 g of the biochar in 1000 mL of orthophosphoric acid (H_3_PO_4_, 0.3 M) and stirring for 30 min at 100 °C, followed by washing with distilled water until pH reaches neutral. Afterwards, the particles were dried in an oven at 100 °C for 30 min before being pyrolyzed in a muffle furnace at 550 °C for 30 min.

Bi_12_O_17_Cl_2_ catalyst was freshly prepared according to the method previously reported by Ma et al.^33^. 1.62 g of bismuth chloride was dissolved in 20 mL of absolute ethanol. The mixture was stirred for 30 min, then 20 mL of 1.2 M NaOH was added to the solution in a drop-wise manner. The mixture was further stirred for 3.0 h and the solid powder was formed and filtered to harvest the catalyst. The obtained Bi_12_O_17_Cl_2_ catalyst powder was dried at a temperature of 60 °C for 6.0 h under vacuum conditions.

To prepare the biochar/Bi_12_O_17_Cl_2_ composite, 0.951 g of Bi_12_O_17_Cl_2_ and 3.0 mg of the prepared biochar were added to 20 mL of absolute ethanol. The mixture was then stirred for 1.0 h at a temperature of 60 °C. Subsequently, the catalyst particles were filtered and dried in an oven at a temperature of 60 °C for 6.0 h under vacuum.

### Experimental details

The experiments were executed in a solar photo-oxidation reactor with parabolic light collectors^34–36^. The details of the reactor and solar spectrum are described by Text S1, Figs. S1 and S2. Firstly, the adsorption performance of the prepared biochar was investigated over 6 h using 100 mg/L of the biochar. Then, the effect of biochar dose was studied by varying the biochar dose from 50 to 200 mg/L under the optimum contact time. Additionally, the photocatalytic performance of bare Bi_12_O_17_Cl_2_ and biochar/Bi_12_O_17_Cl_2_ composite was explored over 6 h using 100 mg/L of both materials. Then, the effect of catalyst dose of the two materials was studied by varying the dose from 50 to 175 mg/L under the optimized time. Additionally, the reusability of the composite was tested via the usage of the same catalyst dose under five repetitive cycles. The catalyst particles were gathered after each run, then dried at room temperature for 24 h prior the following run.

### Analytical methods

The Fourier transfer infrared spectroscopy (FT-IR) data were measured on a Perkin-Elmer FT-IR Spectrophotometer (FT-IR 1650) to specify the functional groups of the synthesized materials. The spectra were recorded in the range of 400–4000 cm^−1^. Scanning electron microscopy (SEM) and energy dispersive X-ray spectroscopy (EDS) analyses were performed to study the morphology and chemical composition of the prepared materials using a JEOL-SEM operating at an accelerating voltage of 20.00 kV equipped with EDS spectrometer. The crystal structure of the prepared materials was explored using an XRD; Bruker-AXS D8, Germany. Further, X-ray photoelectron spectroscopy (XPS) was used to specify the oxidation states and elemental composition of the synthesized materials using XPS, Thermo ScientificTM K-AlphaTM with Al-Kα micro-focused monochromator operated at an energy range up to 4 keV. Additionally, the elemental composition and surface area of the biochar were determined using an X-ray fluorescence (XRF, Axios-Advanced sequential WDXRF spectrometer) and BET surface area (BET Belsorp-max automated), respectively. The surface area of the biochar was measured via the adsorption of nitrogen at 77 K using Brunauer–Emmett–Teller (BET) method and the sample was degassed at 120 °C for 5 h before analysis. Further, the bandgap of bare biochar, pure Bi_12_O_17_Cl_2_ and biochar/Bi_12_O_17_Cl_2_ composite was estimated using UV–visible spectrometer (Jasco, V-630 UV–Vis spectrophotometer) based on Tauc plot method. In Tauc plot method, the relation between photon energy (hv) and (αhv)^0^^.5^ was plotted, where α is the absorption coefficient. The bandgap value is the intersection of the linear portion with the x-axis.

Chemical oxygen demand of samples was determined using the standard method in (Standard Methods for the Examination of Water and Wastewater)^37^. Chemical oxygen demand (COD) of real pharmaceutical wastewater was measured by adding 2.5 mL of the samples to COD bottles which contain 3.5 mL of silver sulfate solution and 1.5 mL of potassium dichromate solution. The silver sulfate solution was prepared by adding 9.5 g of silver sulfate to one liter of sulfuric acid and the solution was left for 1 day without mixing. To prepare the potassium dichromate solution, 10.214 g of dried potassium dichromate was added to 100 mL of distilled water and this solution was added to another solution containing 33.3 g of mercuric sulfate, 167 mL of sulfuric acid and 200 mL of distilled water followed by adding distilled water to reach one Liter. Then, COD bottles were placed in COD reactor for heating the samples at 148 °C for 2 h. After 2 h, the samples were left to chill. A blank sample containing distilled water was placed in the spectrophotometer and autozero was pressed before measuring the COD of the samples. In the case of soluble COD (COD_s_), the samples were filtered by 0.45 µm filter before analysis. The concentration of TC was estimated using a Shimadzu UV–Vis spectrophotometer (Jasco, V-630 UV–Vis spectrophotometer) at a wavelength of 357 nm. The absorption spectra of TC were provided in the supplementary file (Fig. S3). Further, TC concentrations were measured again using high-performance liquid chromatography (HPLC, Shimadzu) as described by Zhang et al. (2022) to confirm the results obtained from UV–Vis spectrophotometer^38^. The HPLC chromatogram of TC was provided in the supplementary file (Fig. S4). The generated intermediates were recognized using liquid chromatography-mass spectroscopy (LC–MS, Shimadzu 2020) using acetonitrile (50%) and water (50%) at a flow rate of 0.2 mL/min.

### Computational modeling

Biochar adsorption mechanism with tetracycline was carried out by computational simulation model i.e. BIOVIA software. Graphite structure was used to have the 3D structure of the biochar derived from Corchorus olitorius on the database.

Probably the graphite could represent the biochar model as reported earlier by^23,39,40^. The surface oxygen groups like COOH, OH and C=O were added to the surface of graphite structure to study the effect of biochar activation on adsorption of tetracycline. 3D structures of tetracycline were downloaded from PubChem and optimized with BIOVIA Forcite before adsorption onto biochar. The binding energy of biochar with tetracycline was estimated using Eq. (1)^41^:

1 $$ \Delta {\text{E}} = {\text{E}}_{{{\text{Biochar}}@{\text{x}}}} {-}{\text{E}}_{{{\text{Biochar}}}} - {\text{E}}_{{\text{x}}} $$

where ΔE: is the binding energy, E_Biochar@x_: is the total energy of complex (biochr@tetracycline), E _Biochar_ is the biochar energy, and E_x_: is the tetracycline energy

### Ethical approval

Experimental research and field studies on *Corchorus olitorius*, including the collection of plant material, complies with relevant institutional, national, and international guidelines and legislation.

## Results and discussion

### Characteristics of Corchorus olitorius-derived biochar

**Figure 1:**
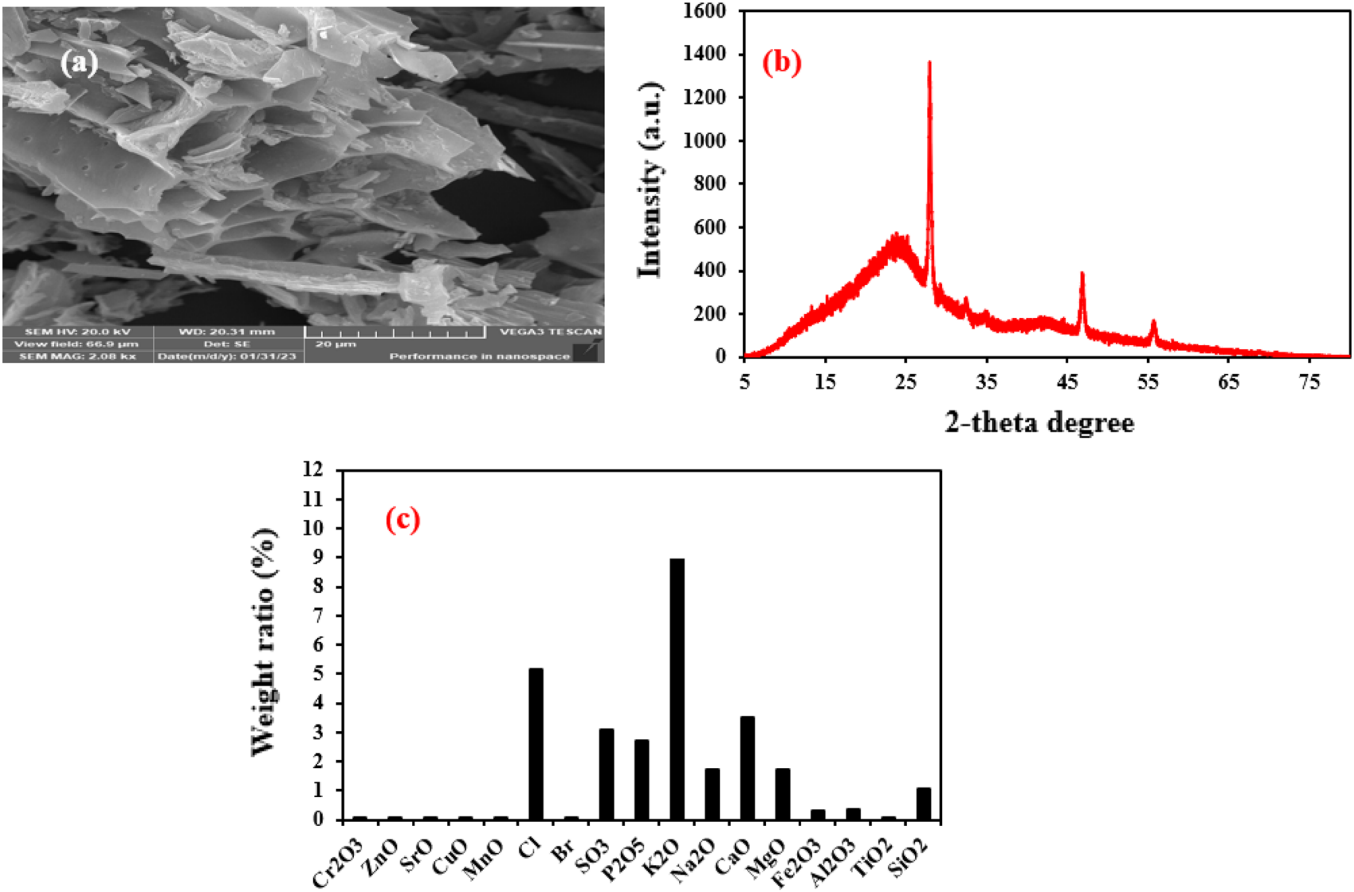
SEM (**a**), XRD (**b**) and XRF (**c**) of the biochar derived from Corchorus olitorius.

Figure 1a shows the SEM image of the prepared biochar indicating that the morphology is vessel-like with high porosity suggesting its high adsorption ability. The diffraction peak between 20° and 30° are imputed to the diffraction plane (002) of the graphite confirming its carbon content as shown in the XRD pattern in Fig. 1b^23^. Additionally, other diffraction peaks are indexed to the metal oxides (e.g., SiO_2_, CaO, MgO) in the prepared biochar detected by XRF as described in Fig. 1c^42^. The sharp peak at 26.7° is attributed to SiO_2_^42^. The XRF analysis of biochar shows the oxides existed in the biochar and their weight ratios as depicted in Fig. 1c. The surface area of the synthesized biochar is 277.6 m^2^/g suggesting the high adsorption performance of the biochar.

### Bi_12_O_17_Cl_2_ catalyst and biochar/Bi_12_O_17_Cl_2_ composite characteristics

**Figure 2:**
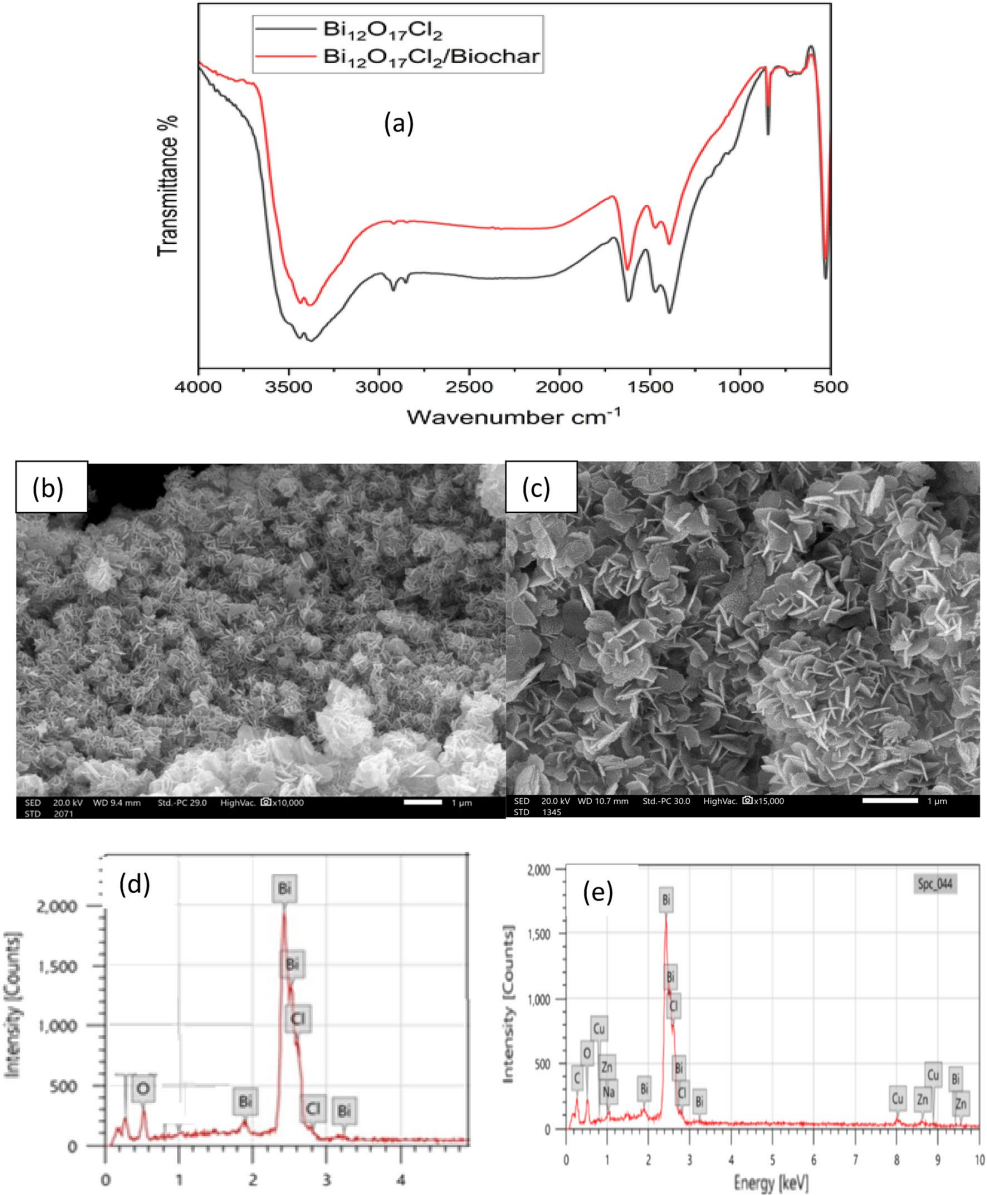
(**a**) Fourier transform infrared spectrum (FTIR) for Bi_12_O_17_Cl_2_ and biochar/Bi_12_O_17_Cl_2_, scanning electron microscope (SEM) for (**b**) Bi_12_O_17_Cl_2_ and (**c**) biochar/Bi_12_O_17_Cl_2_, energy dispersive X-ray spectroscopy (EDX) for (**d**) Bi_12_O_17_Cl_2_ and (e) biochar/Bi_12_O_17_Cl_2_.

The chemical groups in the bare Bi_12_O_17_Cl_2_ and biochar/Bi_12_O_17_Cl_2_ composite were determined by Fourier transform infrared (FT-IR) spectrum as depicted in Fig. 2a. The bands at 3438.57 cm^−1^, (1622.22 cm^−1^, 1469.84 cm^−1^), 1392.82 cm^−1^, 846.4 cm^−1^ and 528.8 cm^−1^ in the case of pristine Bi_12_O_17_Cl_2_ are owing to O–H bond, water molecules, Bi‒Cl stretching vibration, O–Bi–O bending vibration, and Bi=O bending vibration, respectively^24,43^. A slight shift in the bands in the case of biochar/Bi_12_O_17_Cl_2_ to 3435.37 cm^−1^, 1626.56 cm^−1^, 1471.02 cm^−1^, 1393.9 cm^−1^, 846.5 cm^−1^ and 530.3 cm^−1^ was observed due to the incorporation of the biochar.

Scanning electron microscope (SEM) and energy dispersive X-ray spectroscopy (EDX) analyses of the Bi_12_O_17_Cl_2_ and biochar/Bi_12_O_17_Cl_2_ are presented in Fig. 2b–e to show the morphology and chemical constituents of the catalysts. The particle size of pure Bi_12_O_17_Cl_2_ was 39.02 nm (Fig. 2b)_,_ whereas the particle size decreased to 22.799 nm in the case of biochar/Bi_12_O_17_Cl_2_ (Fig. 2c). Further, Fig. 2b,c shows that Bi_12_O_17_Cl_2_ and biochar/Bi_12_O_17_Cl_2_ particles have flower-like structure.

**Figure 3:**
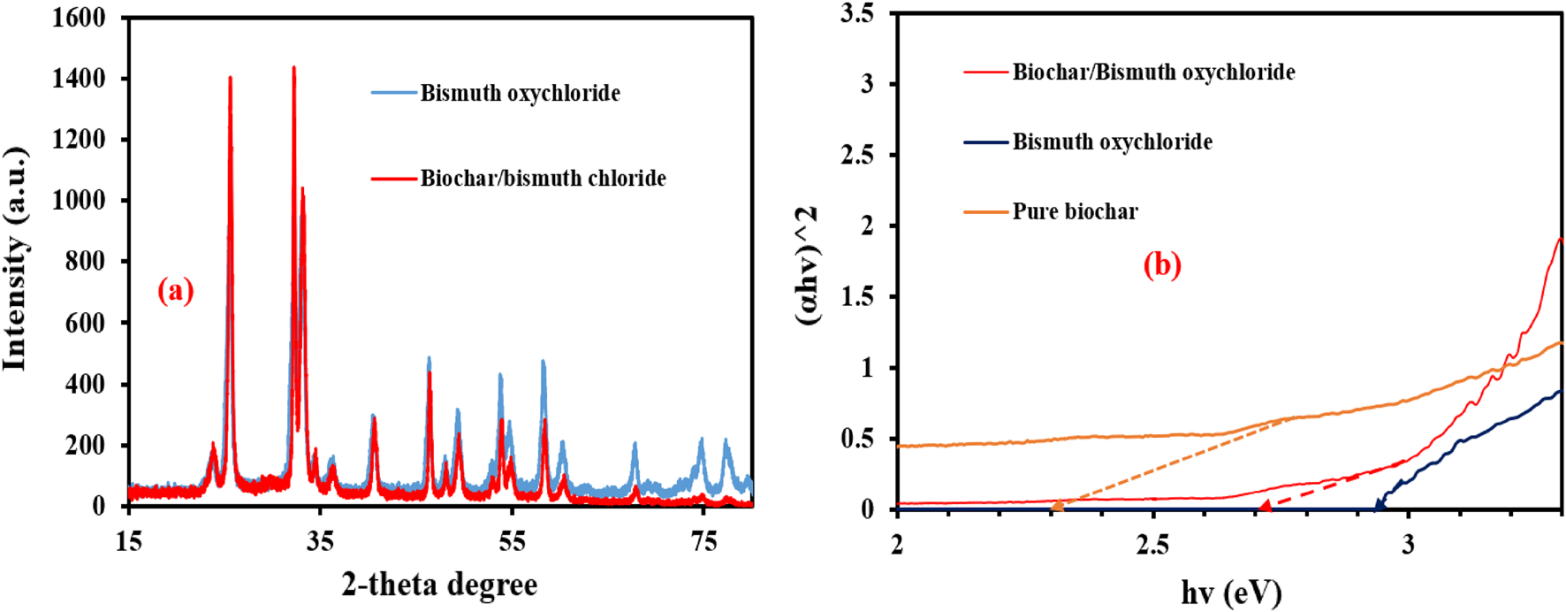
(**a**) XRD patterns and (**b**) bandgap of Bi_12_O_17_Cl_2_ catalyst and biochar/Bi_12_O_17_Cl_2_ composite.

Figure 2d,e depicts the EDX pattern of pure Bi_12_O_17_Cl_2_ and biochar/Bi_12_O_17_Cl_2_ showing that there is no carbon in the case bare Bi_12_O_17_C_l2_, whereas carbon mass ratio was 9.66% in the case of biochar/Bi_12_O_17_Cl_2_ confirming the loading of Bi_12_O_17_Cl_2_ catalyst on the prepared biochar. The mass ratios of Bi in the Bi_12_O_17_Cl_2_ catalyst and Bi_12_O_17_Cl_2_/biochar composite were 70.06 and 65.97%, respectively. The Cl mass ratio was 9.39% in the case of Bi_12_O_17_Cl_2_ catalyst and increased to 10.76 in the case of biochar/Bi_12_O_17_Cl_2_. The mass ratio of oxygen is 8.11% in the case of Bi_12_O_17_Cl_2_ catalyst and 7.55% in the case of biochar/Bi_12_O_17_Cl_2_. The EDX analysis strongly insures the elemental composition of bare Bi_12_O_17_Cl_2_ and biochar/Bi_12_O_17_Cl_2_ composite and the excellent interaction between the biochar and Bi_12_O_17_Cl_2_. The XRD patterns of Bi_12_O_17_Cl_2_ catalyst and Bi_12_O_17_Cl_2_/biochar are illustrated in Fig. 3a. The main peaks observed at 24.01°, 25.86°, 32.49°, 46.4°, 48.1°, 53.8° and 58.3° are imputed to (113), (115), (200), (2012), (220), (315) and (317) diffraction planes of the tetragonal Bi_12_O_17_Cl_2_ (JCPDS No. 37-0702)^44^. In the case of biochar/Bi_12_O_17_Cl_2_, no significant diffraction peaks were observed compared to the XRD pattern in the case of pure Bi_12_O_17_Cl_2_, which might be due to the high dispersion of the catalyst particles on the biochar surface as well as the small content of biochar in the composite^45^. The bandgap of pure biochar was 2.3 eV as given in Fig. 3b. However, the photocatalytic performance of biochar was not high due to low ratios of metal oxides in the prepared biochar. The bandgap of Bi_12_O_17_Cl_2_ catalyst and biochar/Bi_12_O_17_Cl_2_ composite was estimated Fig. 3b as reported by He et al.^29^. The bandgap was 2.9 eV in the case of bare Bi_12_O_17_Cl_2_ and decreased to 2.7 eV in the case of the composite due to the interaction between the biochar and Bi_12_O_17_Cl_2_ phases which could result in the formation of new energy level above the valence band or above the conduction band^20,46^. The reduction of bandgap in the case of the composite confirms the role of biochar to narrow the band gap which improved the utilization of solar light.

**Figure 4:**
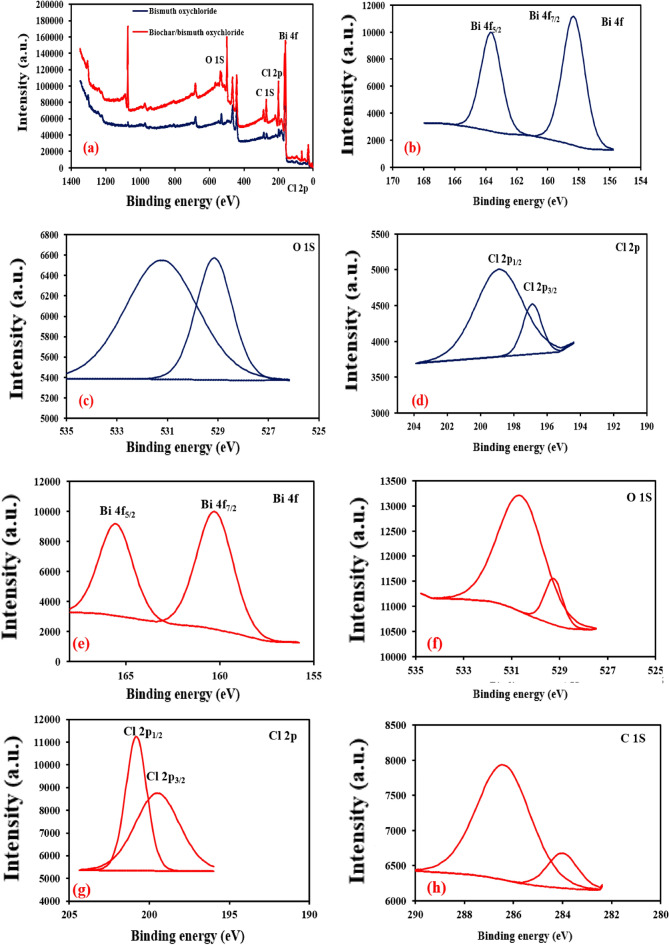
(**a**) XPS survey spectra of Bi_12_O_17_Cl_2_ and biochar/Bi_12_O_17_Cl_2_, (**b**) Bi 4f, (**c**) O 1S and (**d**) Cl 2p spectra of pure Bi_12_O_17_Cl_2_; (**e**) Bi 4f, (**f**) O1S, (**g**) Cl 2p and (**h**) C 1S spectra of biochar/Bi_12_O_17_Cl_2_.

X-ray photoelectron spectroscopy (XPS) analysis for Bi_12_O_17_Cl_2_ and biochar/Bi_12_O_17_Cl_2_ was carried out to show the chemical states of the elements on the catalysts’ surface (Fig. 4). XPS survey in Fig. 4a reaffirmed the growth of Bi, O, Cl and C in the case of biochar/Bi_12_O_17_Cl which confirmed the excellent interaction between the prepared biochar and Bi_12_O_17_Cl.

The peaks with binding energies at about 158.33 and 163.66 eV are assigned to Bi 4f_7/2_ and Bi 4f_5/2_, respectively for Bi_12_O_17_Cl_2_ catalyst, whereas the peaks at 160.28 and 165.56 eV are imputed to Bi 4f_7/2_ and Bi 4f_5/2_, respectively in the case of biochar/Bi_12_O_17_Cl_2_ composite confirming the presence of Bi^3+^ species as shown in Fig. 4b,e^47^. The peaks at 198.82 and 196.93 eV are ascribed to Cl 2p_1/2_ and Cl 2p_3/2_, respectively in the case of pure Bi_12_O_17_Cl_2,_ while the peaks for Cl 2p_1/2_ and Cl 2p_3/2_ are at 199.49 and 200.7 eV, respectively in the case of the composite ensuring the presence of Cl as depicted in Fig. 4d,g^48^. Additionally, the peaks at around 529 eV and 530 eV are imputed to O 1S in Bi-O-Bi bond and O–H bond in the case of Bi_12_O_17_Cl_2_ and biochar/Bi_12_O_17_Cl_2_ (Fig. 4c,f)^4^. Further, the peaks at nearly 287 eV and 284 eV are assigned to C 1S of C–C bond in the biochar in the case of the composite which affirmed the interaction between the components of the composite as given in Fig. 4h^24^.

### Effect of contact time and biochar’s dose on the removal of tetracycline

**Figure 5:**
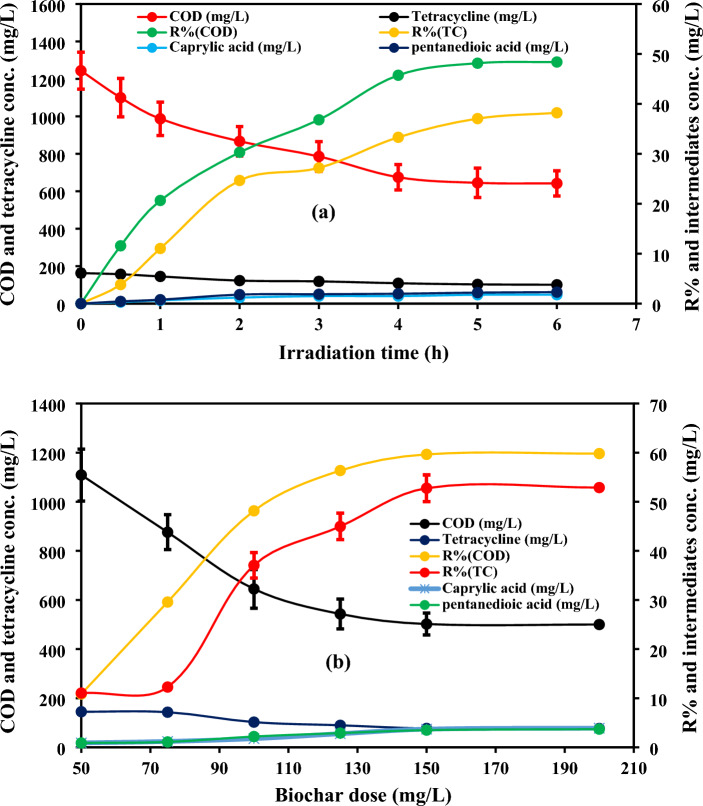
(**a**) Removal of TC and COD by the prepared biochar and (**b**) effect of biochar dose on the removal performance as well as tracking the generated by-products.

Figure 5a shows the removal of TC and the concentration of the generated intermediates over 6 h using 100 mg/L of the prepared biochar at pH 4.7 ± 0.5, initial TC concentration of 163 mg/L and COD of 1244 mg/L. The removal efficiencies of tetracycline and COD were 37% and 48.15%, respectively after 5 h, whereas they were only 38.2% and 48.4% after 6 h indicating that the optimum contact time was 5 h. After 5 h, the biochar’s surface and pores might be saturated by the adsorbed TC molecules^49,50^. Therefore, the removal of TC after 5 h was limited. Additionally, the removal of TC by the biochar could be due to the generated reactive radicals that would be generated as a result of the excitation of metal oxides (e.g., TiO_2_, Fe_2_O_3_, MgO) in the biochar as explained in the XRF analysis^23^. Howbeit, the metal oxides weight ratios are low. Therefore, the removal of biochar was mainly due to adsorption. According to the proposed degradation pathways described in the next sections based on the generated by-products detected by LC–MS, TC could be degraded to simpler by-products such as caprylic acid and pentanedioic acid as a result of the frequent attack by the generated reactive radicals. The concentrations of the generated caprylic acid and pentanedioic acid were 1.77 mg/L and 2.2 mg/L after 5 h, while TC concentration decreased from 163 to 102.6 mg/L at the same time. The low concentration of the generated by-products was due to the modest degradation by the limited number of reactive radicals generated via the illumination of metal oxides on the biochar’s surface which affirmed that most of TC molecules were adsorbed on the biochar’s surface.

Prior optimizing the contact time, different biochar doses (50–200 mg/L) were investigated to specify the optimum biochar dose at a reaction time of 5 h, pH 4.7 ± 0.5, initial TC concentration of 163 mg/L and COD of 1244 mg/L as well as the concentration of the generated intermediates was estimated at different doses as demonstrated in Fig. 5b. The removal efficiencies of COD were 10.8%, 29.5, 48.1%, 56.3%, 59.6% and 59.8%, while the removal ratios of TC were 11%, 12.3%, 37%, 44.9%, 52.7% and 52.9% at biochar doses of 50 mg/L, 75 mg/L, 100 mg/L, 125 mg/L, 150 mg/L and 200 mg/L, respectively. According to the results, the removal efficiency and COD mineralization percentages went up with raising the biochar dose due to the increase of adsorption sites as well as the increase of the generated radicals as a result of the increase of metal oxides. Howbeit, the removal efficiency remained approximately the same with increasing the biochar dose from 150 to 200 mg/L. The biochar particles might agglomerate with increasing the biochar loading above 150 mg/L which could result in the decline of active area, thereby suppressing the expected higher removal at excessive biochar doses as reported by Mensah et al. and Salama et al.^51,52^. The same trend was observed for the generated intermediates, where the concentration of the generated by-products increased from 0.9 to 3.9 mg/L in the case of caprylic acid and from 0.88 to 3.7 mg/L in the case of pentanedioic acid by raising the biochar from 50 to 150 mg/L, respectively, while the concentration of the generated nearly remained the same at biochar dose of 200 mg/L.

### Photodegradation of TC by Bi_12_O_17_Cl_2_ catalyst

**Figure 6:**
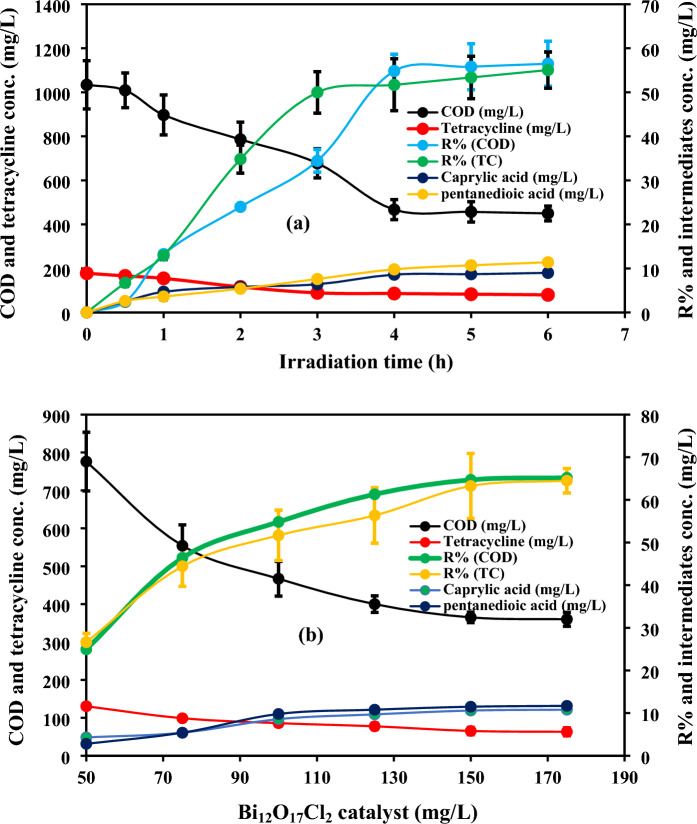
effect of irradiation time on photodegradation of TC over Bi_12_O_17_Cl_2_ with tracking the generated intermediates (**a**) and the degradation performance with tracking the generated intermediates at different Bi_12_O_17_Cl_2_ catalyst doses (**b**).

The degradation of TC and monitoring the concentration of the generated intermediates were attained using 100 mg/L of Bi_12_O_17_Cl_2_ photocatalyst at pH 4.7 ± 0.5, initial TC concentration of 178 mg/L and COD of 1034 mg/L as shown in Fig. 6a. The TC degradation and COD mineralization percentages were 55% and 56.5%, respectively after 6 h, while TC and COD removal efficiencies were 51.7% and 54.8% after 4 h. The slight increase of TC removal efficiency after extending the time to 6 h might be due to the block of active sites with time as a result of the accumulation of TC molecules and its intermediates on the catalyst’s surface which resulted in the decrease of the generated reactive radicals^53^. Additionally, the caprylic acid and pentanedioic acid concentrations were 8.6 mg/L and 9.8 mg/L after 4 h, while they were 9 mg/L and 11.4 mg/L, respectively after 6 h. Therefore, the optimum reaction time was 4 h.

The optimum Bi_12_O_17_Cl_2_ photocatalyst dose was determined by studying the catalyst dose in the range (50–175 mg/L) at pH 4.7 ± 0.5, initial TC concentration of 178 mg/L, reaction time of 4 h and COD of 1034 mg/L as well as the by-products’ concentrations were measured at the different doses (Fig. 6b). The increase of catalyst dose from 50 to 150 mg/L resulted in the raising of TC degradation efficacy from 26.6 to 63% and COD mineralization ratio from 24.9 to 64.7%, while TC degradation ratio and COD mineralization percentage were 64.5% and 65.2% at a catalyst dose of 175 mg/L. Raising the catalyst dose generally improved the degradation performance due to the increase of active sites which could lead to the generation of more radicals, thereby enhancing the degradation efficiency^54^. On the contrary, the rising of catalyst dose above 150 mg/L did not show a significant change in the degradation performance, as excessive increase of the catalyst dose could raise the turbidity of the solution which might result in the scattering of photons and the inactivation of most of the active sites^30^. Additionally, the agglomeration between the catalyst particles might increase in the case of elevating the catalyst dose which reduced the active sites leading to the inhibition of the accelerated degradation performance^55^. Regarding the by-products’ concentrations, the caprylic acid and pentanedioic acid concentrations were 10.6 mg/L and 11.5 mg/L at a catalyst dose of 150 mg/L, whereas they were 10.8 mg/L and 11.7 mg/L, respectively at a catalyst dose of 175 mg/L. Thus, the optimum catalyst dose was 150 mg/L.

### Photodegradation of TC by biochar/Bi_12_O_17_Cl_2_ composite

**Figure 7:**
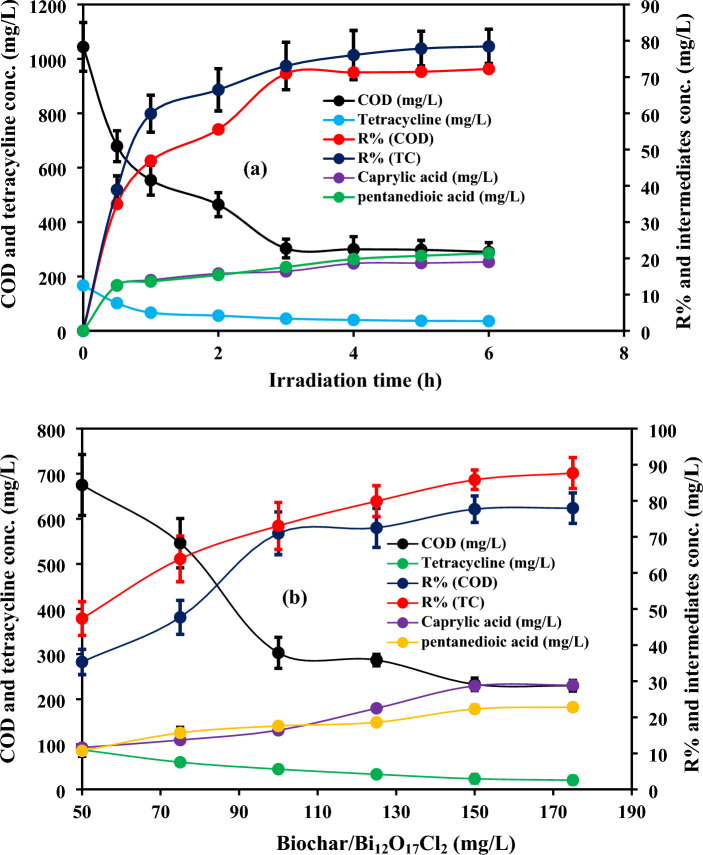
(**a**) Degradation of TC using the prepared composite and the concentration of the generated by-products with time and (**b**) effect of biochar/Bi_12_O_17_Cl_2_ dose on the degradation performance and the concentration of the formed intermediates under different doses.

The degradation of TC and the concentration of the generated intermediates were studied at a composite dose of 100 mg/L, pH 4.7 ± 0.5, initial TC concentration of 167 mg/L, reaction time of 6 h and COD of 1044 mg/L as shown in Fig. 7a. TC removal efficiency was 73% after 3 h, whereas it was only 78.4% by prolonging the time to 6 h. Further, COD mineralization ratios were 70.9% and 72.2% after 3 h and 6 h, respectively. Additionally, the caprylic acid and pentanedioic acid concentrations increased to 16.4 mg/L and 17.6 mg/L after 3 h and they were 19 mg/L and 21.4 mg/L, respectively after 6 h. According to the results, the optimum reaction time could be 3 h. As we explained in the above section, the degradation and mineralization ratios did not greatly change after extending the time due to the accumulated TC molecules and its intermediates on the composite’s surface which blurred the active sites. The degradation efficiencies and mineralization ratios were higher in the case of the prepared hybrid than that of pure Bi_12_O_17_Cl_2_ under a shorter reaction time which could participate in reducing the treatment cost. The added biochar could accept electrons which improved the separation efficiency between electrons and holes. Additionally, the enhanced performance might be owing to the combined effects of adsorption and photodegradation.

The optimum composite dose was specified using various doses (50, 75, 100, 125, 150 and 175 mg/L) as well as the by-products’ concentrations were specified at these values at pH 4.7 ± 0.5, initial tetracycline concentration of 167 mg/L, reaction time of 3 h and COD of 1044 mg/L as given in Fig. 7b. The increase of composite dose to 150 mg/L raised the degradation efficiency of TC to 85.8% and COD mineralization ratio to 77.7% due to the increase of the generated radicals with elevating the catalyst dose. Howbeit, the increase of catalyst dose to 175 mg/L did not improve the degradation efficiency due to the aforementioned explanations (light scattering and particle agglomeration). As a result, the caprylic acid and pentanedioic acid concentrations were 28.7 mg/L and 22.3 mg/L at catalyst dose of 150 mg/L, whereas their concentrations nearly remained the same in the case of raising the composite dose to 175 mg/L. Therefore, the optimum composite dose was 150 mg/L. The electrical energy consumption under the optimal conditions was measured based on the equation reported by Vaiano et al.^56^. The electrical energy consumption in this system is around 8 KWh/m^3^. Further, the obtained results under the optimal conditions were compared with the results in the previous studies as shown in Table S1 to evaluate the performance of our system compared to other systems. Most of the studies performed their experimental work on synthetic wastewater at low concentrations of tetracycline. However, in our study, the degradation experiments were conducted on real pharmaceutical wastewater with high concentrations of tetracycline. Real pharmaceutical wastewater is a complex matrix that may contain other pollutants compete with the main pollutant and can be degraded by the reactive radicals. However, according to the comparison in Table S1, the degradation performance of our system was high and was not affected by the complexity of real wastewater.

### Reusability of the prepared composite

**Figure 8:**
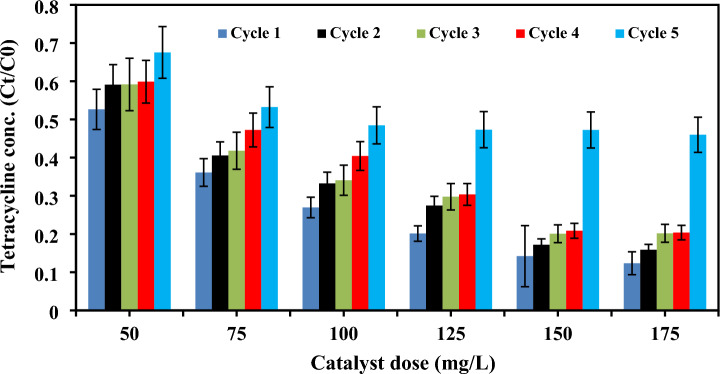
Reusability of the prepared composite at different doses in five succeeding cycles.

Assessing the recyclability of the prepared composite is essential to confirm the stability of the composite under a prolonged reaction time prior the full-scale application. Additionally, using the catalyst in successive cycles could reduce the treatment cost. To evaluate the reusability of the composite, the composite was tested under five succeeding cycles using different composite doses (50–175 mg/L) at pH 4.7 ± 0.5, initial TC concentration of 167 mg/L and reaction time of 3 h., as given in Fig. 8. The degradation efficiency of TC was 85.8% in the first cycle and decreased to 52.7% is the fifth cycle in the case of composite dose of 150 mg/L, while the degradation ratio was 79.2% after the fourth cycle indicating the stability of the catalyst under four cycles. In the case of all doses, the degradation percentage started to greatly decrease after the fourth cycle. Thus, the prepared catalyst could be efficiently reused under four cycles. The decrease of degradation efficiencies with cycles might be due to the loss of catalyst particles during samples withdrawal. Moreover, the active sites might be blocked by the pollutant molecules and its intermediates which resulted in the suppression of radicals’ generation.

### Degradation mechanism and proposed degradation pathways

**Figure 9:**
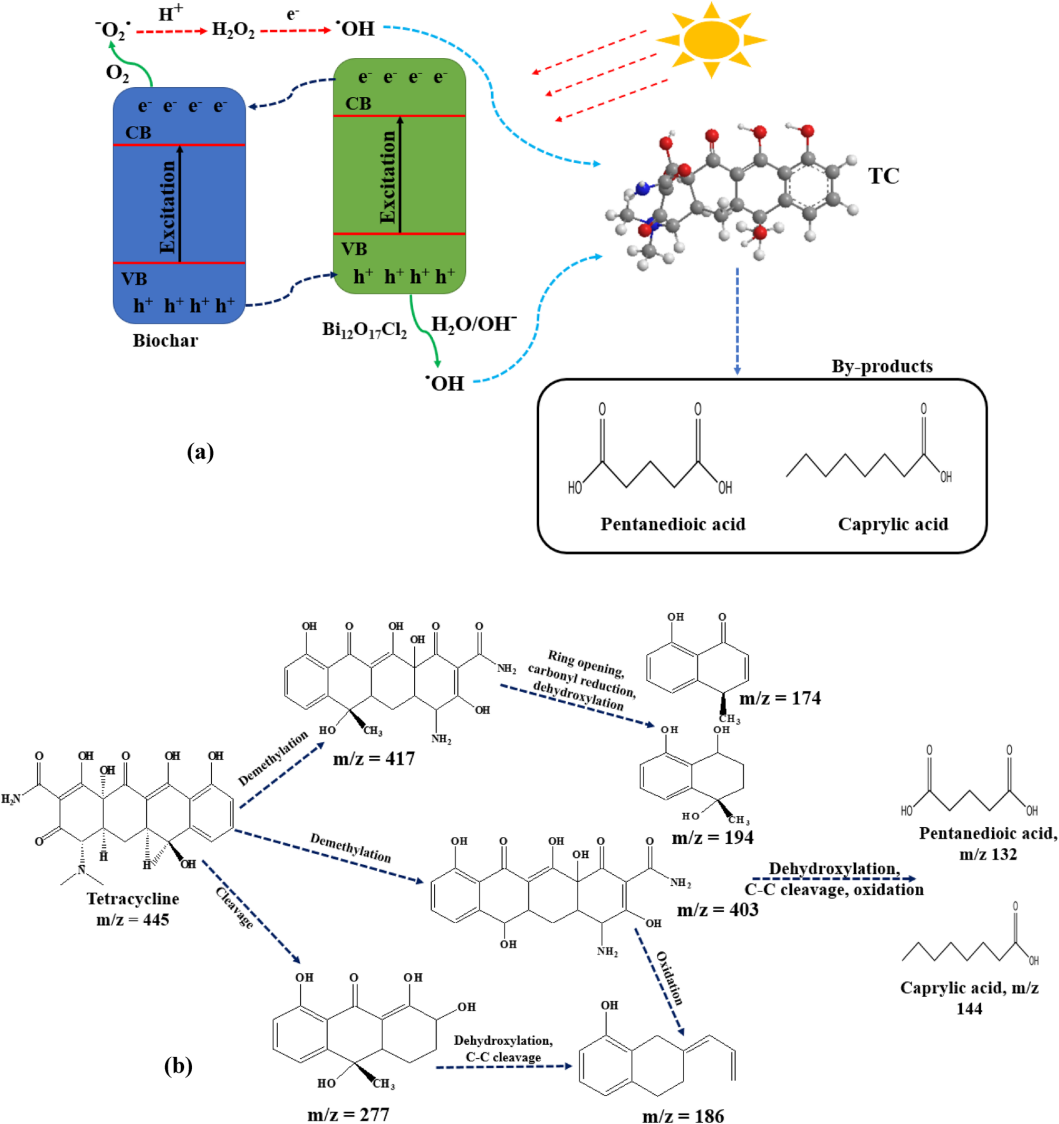
(**a**) Degradation mechanism of TC over biochar/Bi_12_O_17_Cl_2_ and (**b**) proposed degradation pathways.

Prior the excitation of the composite, electrons and holes could be generated in the biochar and Bi_12_O_17_Cl_2_, then the electrons in the conduction band (CB) of Bi_12_O_17_Cl_2_ started to move the CB of the biochar and the holes in the valence band (VB) of the biochar transferred to the VB of Bi_12_O_17_Cl_2_ which suppressed the recombination between charge carriers as shown in Fig. 9a. Then, the electrons and holes could react with oxygen and hydroxyl ions, respectively to form superoxide radicals and hydroxyl radicals, respectively. Then, the generated superoxide radicals could react with H^+^ to form H_2_O_2_ which could further react with electrons to generate additional hydroxyl radicals^24^. For the bare Bi_12_O_17_Cl_2_, the electrons and holes could recombine rapidly. Thus, the composite showed higher performance. The continuous oxidation of TC by the generated radicals could convert TC to simpler intermediates such as Caprylic acid and pentanedioic acid.

The generated intermediates were identified using LC–MS and the degradation pathways were proposed according to the generated reactive radicals and their reaction with TC as shown in Fig. S5. The degradation of TC (m/z 445, Fig. S5a) could start with the demethylation of TC to produce two by-products with m/z 403 and 417 (Fig. S5b,c)^57^. Additionally, the aromatic ring in TC could be cleaved to produce another intermediate with m/z 277 (Fig. S5b)^58^. Then, the dehydroxylation, oxidation and C–C bond cleavage in the intermediates with m/z 277 and 403 could result in the formation of an intermediate with m/z 186 (Fig. S5b)^58^. Additionally, the by-product with m/z 417 could be further attacked by the generated radicals leading to ring opening, dehydroxylation and carbonyl reduction, thereby forming the intermediates with m/z 174 and 194 (Fig. S5a)^57^. Then, the by-products with m/z 186, 174 and 194 could be further degraded to simpler by-products with m/z 144 (caprylic acid) and 132 (pentanedioic acid) (Fig. S5b,c) via the attack of the generated radical, dehydroxylation and cleavage of C–C bonds. Figure 9b depicts the suggested degradation pathways.

### Computational modeling

**Figure 10:**
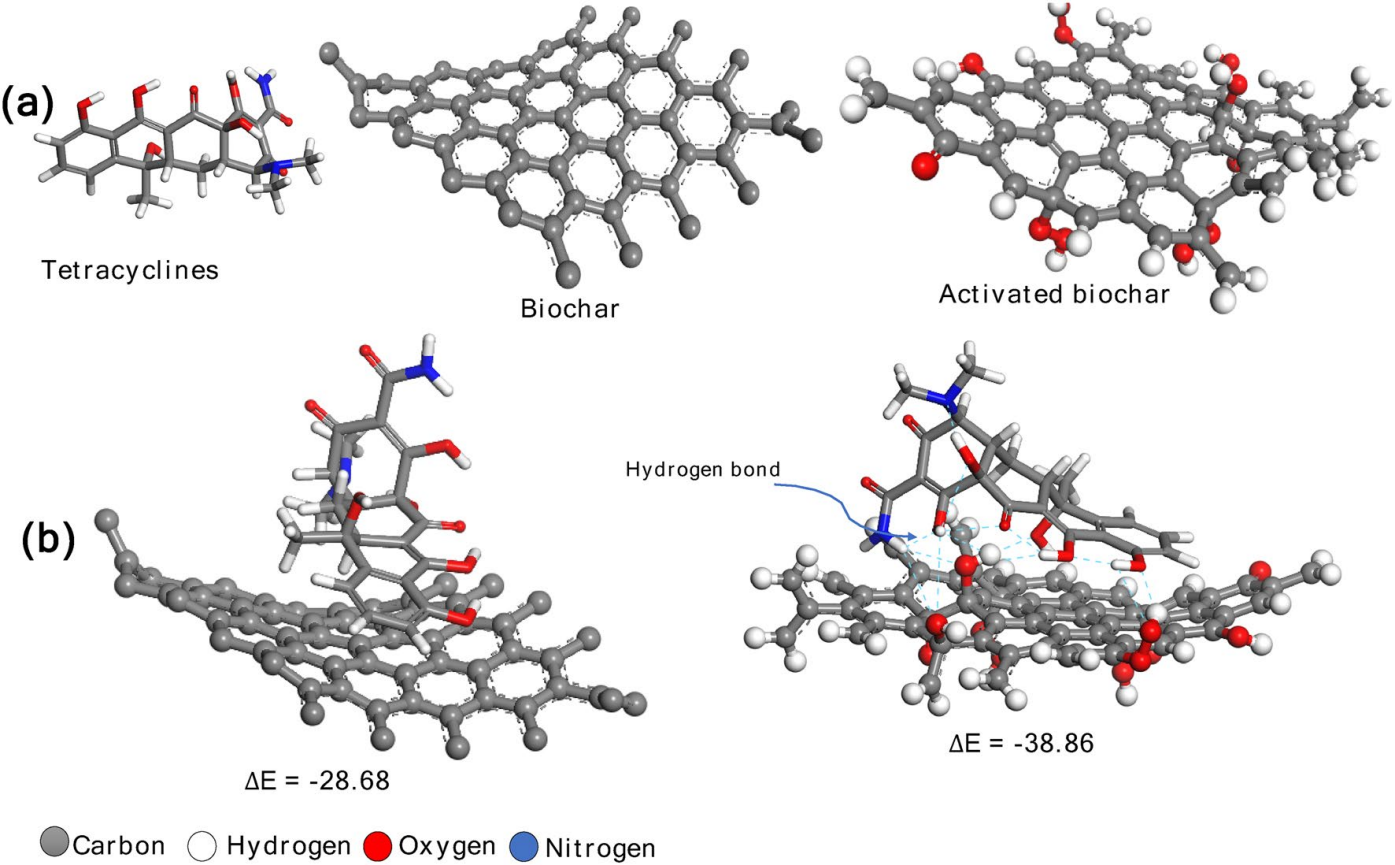
(**a**) The geometry optimized structure of Tetracycline, biochar, and surface activated biochar. (**b**) The adsorption of tetracycline on the surface of the biochar and surface activated biochar.

The adsorption locator module in BIOVIA was used to quantify the adsorption energy between the prepared biochar (adsorbent) and tetracycline (adsorbate) and to study the effect of surface activation of biochar on the binding energy (Fig. 10), as reported earlier^23,59^. According to the computational modeling results, biochar exhibited adsorption energy of − 28.68 kJ/mol, while surface activated biochar showed adsorption energy of − 38.86 kJ/mol to capture tetracycline. The adsorption energy increase in the case of activated biochar was due to the presence of numerous surface oxygen groups such as COOH, OH and C=O on the activated biochar surface. Also, in case of activated biochar, hydrogen bond was observed between H and O in tetracycline and the surface oxygen group of activated biochar.

### Conclusions

XRD, EDX, XPS and FTIR analyses affirmed the excellent fabrication of biochar/Bi_12_O_17_Cl_2_ composite. The activation of biochar ameliorated the adsorption energy to capture TC. The optimum biochar dose was 150 mg/L, where the removal efficiencies of TC and COD were 52.7% and 59.6%, respectively within 5 h, while the degradation and mineralization ratios went up to 63% and 64.7%, respectively using 150 mg/L of pristine Bi_12_O_17_Cl_2_. Biochar/Bi_12_O_17_Cl_2_ showed higher degradation performance compared to the individual components due to the capability of the biochar to accept electrons which improved the separation efficiency between charge carriers. The prepared composite could be reused for four cycles efficiently, where the degradation efficiency was 85.5% in the first cycle and reduced to 79.2% in the fourth cycle. TC could be mineralized to caprylic acid and pentanedioic acid via oxidation, dehydroxylation, demethylation, ring opening and C–C cleavage. The efficient degradation performance, stability and low-cost provide the synthesized catalyst a superiority to be utilized in large-scale reactors for the mineralization of real industrial effluents.

### Supplementary Information

Supplementary Information.

## Data Availability

The datasets used and/or analyzed during the current study is available with the corresponding author up on request.

## References

[1] Guo J (2021). Photocatalytic degradation of tetracycline antibiotics using delafossite silver ferrite-based Z-scheme photocatalyst: Pathways and mechanism insight. Chemosphere.

[2] Samy M, Mensah K, El-Fakharany EM, Elkady M, Shokry H (2023). Green valorization of end-of-life toner powder to iron oxide-nanographene nanohybrid as a recyclable persulfate activator for degrading emerging micropollutants. Environ. Res..

[3] Allam A, Fleifle A, Tawfik A, Yoshimura C, El-Saadi A (2015). A simulation-based suitability index of the quality and quantity of agricultural drainage water for reuse in irrigation. Sci. Total Environ..

[4] Jiang Q (2023). Construction of 2D/2D Bi_12_O_17_Cl_2_/Zn-HMT Z-scheme heterojunction for efficient photocatalytic tetracycline hydrochloride degradation and Cr(VI) reduction. J. Alloys Compd..

[5] Tawfik A, El-Gohary F, Ohashi A, Harada H (2006). The influence of physical-chemical and biological factors on the removal of faecal coliform through down-flow hanging sponge (DHS) system treating UASB reactor effluent. Water Res..

[6] Wang C, Yan R, Cai M, Liu Y, Li S (2023). A novel organic/inorganic S-scheme heterostructure of TCPP/Bi_12_O_17_Cl_2_ for boosting photodegradation of tetracycline hydrochloride: Kinetic, degradation mechanism, and toxic assessment. Appl. Surf. Sci..

[7] Tawfik A, Klapwijk B, Van Buuren J, El- Gohary F, Lettinga G (2004). Physico-chemical factors affecting the *E. coli* removal in a rotating biological contactor (RBC) treating UASB effluent. Water Res..

[8] Chen J, Zhong J, Li J, Qiu K (2021). Boosted photocatalytic removal of tetracycline on S-scheme Bi_12_O_17_C_l2_/α-Bi_2_O_3_ heterojunctions with rich oxygen vacancies. Appl. Surf. Sci..

[9] Tawfik A (2022). Solar photo-oxidation of recalcitrant industrial wastewater: A review. Environ. Chem. Lett..

[10] El-Gohary F, Tawfik A, Badawy M, El-Khateeb MA (2009). Potentials of anaerobic treatment for catalytically oxidized olive mill wastewater (OMW). Bioresour. Technol..

[11] Chiu Y-H, Chang T-FM, Chen C-Y, Sone M, Hsu Y-J (2019). Mechanistic insights into photodegradation of organic dyes using heterostructure photocatalysts. Catalysts.

[12] Elsharkawy K (2020). Paperboard mill wastewater treatment via combined dark and LED-mediated fermentation in the absence of external chemical addition. Bioresour. Technol..

[13] Samy M, Alalm MG, Mossad M (2020). Utilization of iron sludge resulted from electro-coagulation in heterogeneous photo-fenton process. Water Pract. Technol..

[14] Mishra N (2017). A review on advanced oxidation processes for effective water treatment. Curr. World Environ..

[15] Samy M, Mensah K, Alalm MG (2022). A review on photodegradation mechanism of bio-resistant pollutants : Analytical methods, transformation products, and toxicity assessment. J. Water Process Eng..

[16] Ashrafi P (2023). A detailed electrochemical study of anti-malaria drug hydroxychloroquine: Application of a highly porous 3D multi-metal oxide carbon felt/β-PbO2-ZrO_2_−MoOx electrode for its electrocatalytic degradation. Electrochim. Acta.

[17] Rahmani A (2023). The integration of PbO_2_-based EAOPs with other advanced oxidation processes for improved treatment of water and wastewater. Curr. Opin. Electrochem..

[18] Asgari G (2023). Mineralization and biodegradability improvement of textile wastewater using persulfate/dithionite process. Biomass Convers. Biorefinery.

[19] Belver C, Rodriguez JJ, Bedia J (2020). Degradation pathways of emerging contaminants using TiO_2_-activated carbon heterostructures in aqueous solution under simulated solar light. Chem. Eng. J..

[20] Samy M (2020). Innovative photocatalytic reactor for the degradation of chlorpyrifos using a coated composite of ZrV_2_O_7_ and graphene nano-platelets. Chem. Eng. J..

[21] Rahmani A, Shabanloo A, Shabanloo N (2023). A mini-review of recent progress in lead dioxide electrocatalyst for degradation of toxic organic pollutants. Mater. Today Chem..

[22] Guo Q, Zhou C, Ma Z, Yang X (2019). Fundamentals of TiO_2_ photocatalysis: Concepts, mechanisms, and challenges. Adv. Mater..

[23] Samy M (2023). Treatment of hazardous landfill leachate containing 1,4 dioxane by biochar-based photocatalysts in a solar photo-oxidation reactor. J. Environ. Manage..

[24] Zhou C (2018). Rational design of carbon-doped carbon nitride/Bi_12_O_17_Cl_2_ composites: A promising candidate photocatalyst for boosting visible-light-driven photocatalytic degradation of tetracycline. ACS Sustain. Chem. Eng..

[25] Chang F (2019). Ag/AgCl nanoparticles decorated 2D-Bi_12_O_17_Cl_2_ plasmonic composites prepared without exotic chlorine ions with enhanced photocatalytic performance. Mol. Catal..

[26] Quan Y, Wang B, Liu G, Li H, Xia J (2021). Carbonized polymer dots modified ultrathin Bi_12_O_17_Cl_2_ nanosheets Z-scheme heterojunction for robust CO_2_ photoreduction. Chem. Eng. Sci..

[27] Zhou C (2018). In situ grown AgI/Bi_12_O_17_Cl_2_ heterojunction photocatalysts for visible light degradation of sulfamethazine: Efficiency, pathway, and mechanism. ACS Sustain. Chem. Eng..

[28] Wang L, Min X, Sui X, Chen J, Wang Y (2020). Facile construction of novel BiOBr/Bi_12_O_17_Cl_2_ heterojunction composites with enhanced photocatalytic performance. J. Colloid Interface Sci..

[29] He G (2015). Facile synthesis of flower-like Bi_12_O_17_Cl_2_/β-Bi_2_O_3_ composites with enhanced visible light photocatalytic performance for the degradation of 4-tert-butylphenol. Appl. Catal. B Environ..

[30] Samy M (2022). Solar-light-driven ZnO/biochar treatment of pesticides contaminated wastewater: A practical and computational study. Energy Sci. Eng..

[31] Bhavani P, Hussain M, Park Y-K (2022). Recent advancements on the sustainable biochar based semiconducting materials for photocatalytic applications: A state of the art review. J. Clean. Prod..

[32] Dey T, Bhattacharjee T, Nag P, Ghati A, Kuila A (2021). Bioresource technology reports valorization of agro-waste into value added products for sustainable development. Bioresour. Technol. Rep..

[33] Ma J (2020). Fabrication of graphene/Bi_12_O_17_Cl_2_ as an effective visible-light photocatalyst. Mater. Res. Bull..

[34] Gar Alalm M, Ookawara S, Fukushi D, Sato A, Tawfik A (2016). Improved WO_3_ photocatalytic efficiency using ZrO_2_ and Ru for the degradation of carbofuran and ampicillin. J. Hazard. Mater..

[35] Alalm MG, Tawfik A, Ookawara S (2015). Combined solar advanced oxidation and PAC adsorption for removal of pesticides from industrial wastewater. J. Mater. Environ. Sci..

[36] Gar Alalm M, Tawfik A, Ookawara S (2016). Solar photocatalytic degradation of phenol by TiO_2_/AC prepared by temperature impregnation method. Desalin. Water Treat..

[37] APHA. Standard Methods for the Examination of Water and Wastewater. *23th ed. Washington, DC Am. Public Heal. Assoc. Water Work. Assoc. Water Environ. Fed.* (2017).

[38] Zhang Y (2022). Insight in degradation of tetracycline in mariculture wastewater by ultraviolet/persulfate advanced oxidation process. Environ. Res..

[39] Chen Q, Zheng J, Xu J, Dang Z, Zhang L (2019). Insights into sulfamethazine adsorption interfacial interaction mechanism on mesoporous cellulose biochar: Coupling DFT/FOT simulations with experiments. Chem. Eng. J..

[40] Fan Y (2021). Adsorption of sulfonamides on biochars derived from waste residues and its mechanism. J. Hazard. Mater..

[41] Xu Y (2022). Desert beetle-like microstructures bridged by magnetic Fe_3_O_4_ grains for enhancing oil-in-water emulsion separation performance and solar-assisted recyclability of graphene oxide. Chem. Eng. J..

[42] Liu Y, Zhao X, Li J, Ma D, Han R (2012). Characterization of bio-char from pyrolysis of wheat straw and its evaluation on methylene blue adsorption. Desalin. Water Treat..

[43] Shi T (2022). Construction of interface electric field by electrostatic self-assembly: Enhancing the photocatalytic performance of 2D/2D Bi_12_O_17_Cl_2_/g-C_3_N_4_ nanosheets. J. Mater. Sci. Mater. Electron..

[44] Chang F (2019). Oxygen-rich bismuth oxychloride Bi_12_O_17_Cl_2_ materials: Construction, characterization, and sonocatalytic degradation performance. Ultrason. Sonochem..

[45] Ge L, Han C, Xiao X, Guo L (2013). Synthesis and characterization of composite visible light active photocatalysts MoS_2_-g-C_3_N_4_ with enhanced hydrogen evolution activity. Int. J. Hydrogen Energy.

[46] Samy M (2021). CNTs/MOF-808 painted plates for extended treatment of pharmaceutical and agrochemical wastewaters in a novel photocatalytic reactor. Chem. Eng. J..

[47] Hao L, Huang H, Guo Y, Du X, Zhang Y (2017). Bismuth oxychloride homogeneous phasejunction BiOCl/Bi 12 O 17 Cl 2 with unselectively efficient photocatalytic activity and mechanism insight. Appl. Surf. Sci..

[48] Zheng J (2018). A visible-light-driven heterojuncted composite WO_3_/Bi_12_O_17_Cl_2_: Synthesis, characterization, and improved photocatalytic performance. J. Colloid Interface Sci..

[49] Mensah K, Samy M, Mahmoud H, Fujii M, Shokry H (2022). Rapid adsorption of sulfamethazine on mesoporous graphene produced from plastic waste: Optimization, mechanism, isotherms, kinetics, and thermodynamics. Int. J. Environ. Sci. Technol..

[50] Samy M (2022). Novel biosynthesis of graphene-supported zero-valent iron nanohybrid for efficient decolorization of acid and basic dyes. Sustain.

[51] Mensah K, Mahmoud H, Fujii M, Samy M, Shokry H (2022). Dye removal using novel adsorbents synthesized from plastic waste and eggshell: Mechanism, isotherms, kinetics, thermodynamics, regeneration, and water matrices. Biomass Convers. Biorefinery.

[52] Salama E (2022). The superior performance of silica gel supported nano zero-valent iron for simultaneous removal of Cr (VI). Sci. Rep..

[53] Khairnar SD (2018). Hydrothermally synthesized nanocrystalline Nb_2_O_5_ and its visible-light photocatalytic activity for the degradation of congo red and methylene blue. Iran. J. Catal..

[54] Hydrothermally U (2012). Photocatalytic degradation of municipal wastewater and brilliant blue dye photocatalytic degradation of municipal wastewater and brilliant blue dye using hydrothermally synthesized surface-modified silver-doped ZnO designer particles. Int. J. Photoenergy.

[55] Samy M, Ibrahim MG, Alalm MG, Fujii M (2019). Effective photocatalytic degradation of sulfamethazine by CNTs/LaVO 4 in suspension and dip coating modes. Sep. Purif. Technol..

[56] Vaiano V, Sacco O, Sannino D (2019). Electric energy saving in photocatalytic removal of crystal violet dye through the simultaneous use of long-persistent blue phosphors, nitrogen-doped TiO_2_ and UV-light emitting diodes. J. Clean. Prod..

[57] Zhang N, Chen J, Fang Z, Tsang EP (2019). Ceria accelerated nanoscale zerovalent iron assisted heterogenous Fenton oxidation of tetracycline. Chem. Eng. J..

[58] Xin S (2021). Enhanced heterogeneous photo-Fenton-like degradation of tetracycline over CuFeO_2_/biochar catalyst through accelerating electron transfer under visible light. J. Environ. Manage..

[59] Younes HA, Taha M, Mahmoud R, Mahmoud HM, Abdelhameed RM (2022). High adsorption of sodium diclofenac on post-synthetic modified zirconium-based metal-organic frameworks: Experimental and theoretical studies. J. Colloid Interface Sci..

