# Supplementary material for: A novel Corchorus olitorius-derived biochar/Bi12O17Cl2 photocatalyst for decontamination of antibiotic wastewater containing tetracycline under natural visible light
Source: Sci Rep. 2023 Aug 14;13:13190. doi: 10.1038/s41598-023-38715-4 (PMC10425469; doi:10.1038/s41598-023-38715-4)
Supplement: Supplementary file 1 — Supplementary Information. [file 41598_2023_38715_MOESM1_ESM.docx]

**A novel Corchorus olitorius-derived biochar/Bi_12_O_17_Cl_2_ photocatalyst for decontamination of antibiotic wastewater containing tetracycline under natural visible light**

**Mahmoud Samy^1^, Mohamed Gar Alalm^1^, Ribh S. abodlal^2^, Ali El-Dissouky^2^, Mohamed N. Khalil^3^, Ehab R El-Helow^4^, Tarek El-Sayed Khalil^2^, Ahmed Tawfik^3^[[1]](#footnote-1)^©^**

^1^ Public Works Engineering Department, Faculty of Engineering, Mansoura University, Mansoura 35516, Egypt

^2^Chemistry Department, Faculty of Science, Alexandria University, Alexandria, Egypt

^3^Water Pollution Research Department, National Research, Centre, P.O. Box 12622, Giza, Egypt

^4^Department of Botany and Microbiology, Faculty of Science, Alexandria University,

The 1^st^, 3^rd^ and 8^th^ author is equally contributed

**Supplementary file:**

**Text S1:**

The photo-oxidation of TC was performed using the reactor in Fig. S1 that was constructed in Borg El Arab City, Egypt (Latitude 30°52’, Longitude 29°35’). The polluted solution with a volume of 2.2 L was recirculated passing through six borosilicate tubes (diameter = 2.54 cm, length = 75 cm and area = 0.36 m^2^) via a pump. The tubes were fixed on a curved aluminum reflector with radii of 9.2 cm with an inclination angle of 30° to utilize the largest portion of solar light and the presence of aluminum reflector confirms the illumination of the total area of the tubes by solar light. The solar spectrum is provided in Fig. S2. The tubes are made of borosilicate, and they are transparent to allow the passage of UV and visible light. The first 30 min of the reaction were performed without light (Dark) to achieve equilibrium of the adsorbed pollutant molecules on the catalyst’s surface. Normalization of the illumination time in all experiments to UV intensity of 30 w/m^2^ was conducted according to eqs. (1, 2)

$$t_{30w,n}= t_{30w,n-1}+ {\Delta t}_{n}\left( \frac{UV}{30} \right)\left( \frac{V_{i}}{V_{t}} \right) (1)$$

${\Delta t}_{n}= t_{n}-t_{n-1} (2)$

where t_n_: the experimental reaction time; UV: the average solar ultraviolet radiation (W/m^2^) during a period of ∆t_n_, t_30w_: the normalized illumination time which refers to a constant solar UV power of 30 W/m^2^ (typical solar UV power on a perfectly sunny day around noon), V_t_: the total reactor volume and V_i_: the total irradiated volume.


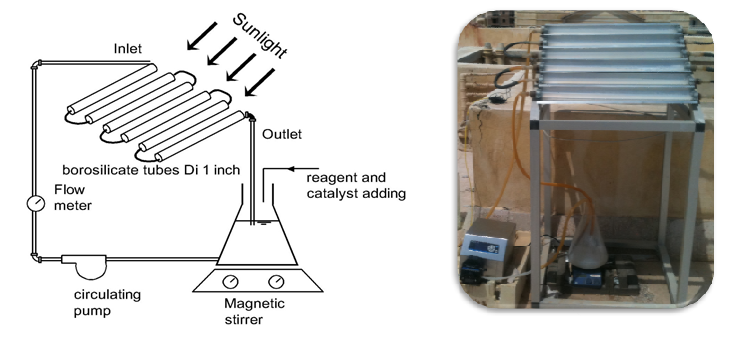


**Fig. S1.** Details of the photo reactor


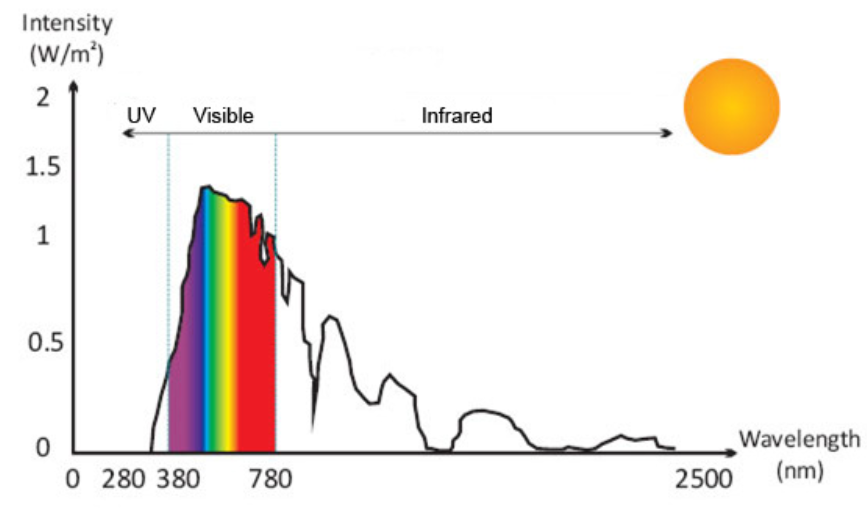


**Fig. S2.** Solar spectrum ^1^.


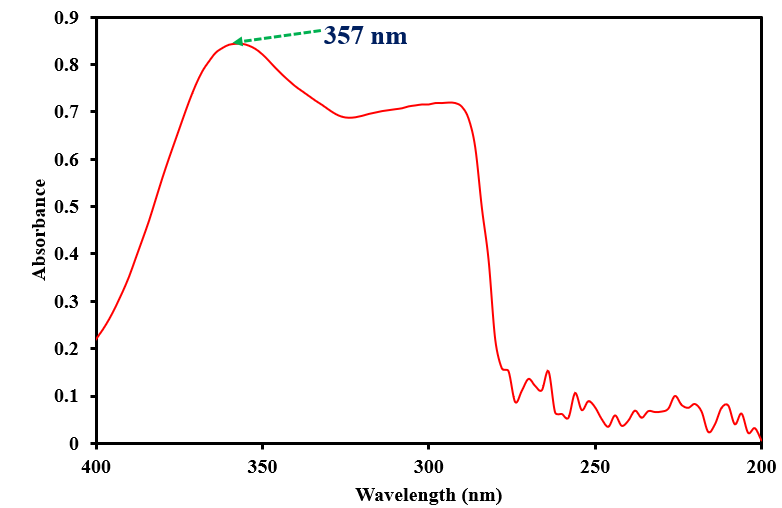


**Fig. S3.** Absorption spectra of TC


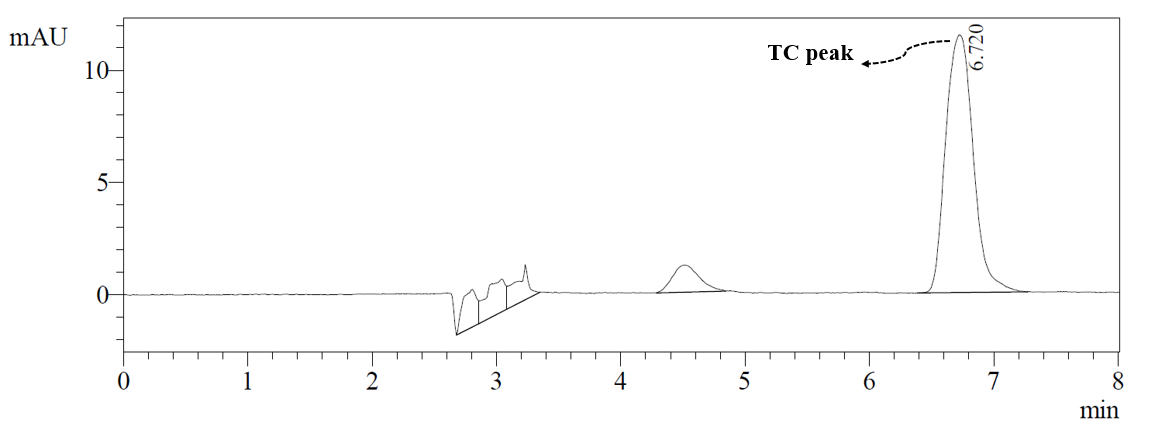


**Fig. S4.** HPLC chromatogram of TC.


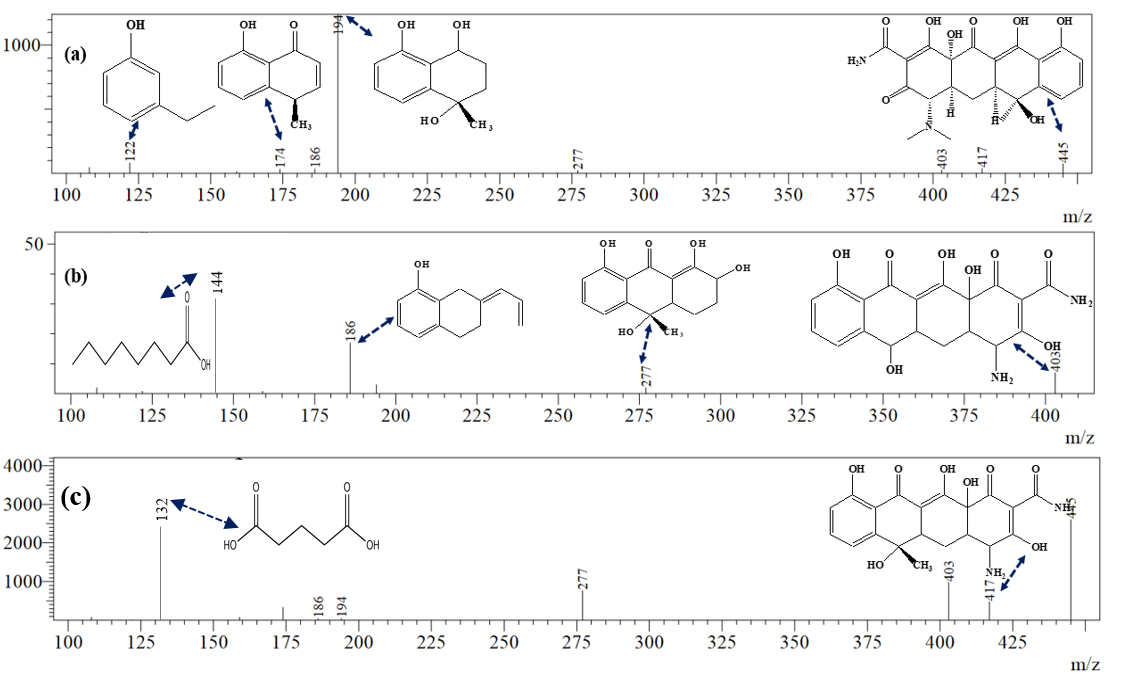


**Fig. S5.** m/z signal peaks of the generated by-products.

**Table S1** Comparison with the previous studies for the degradation of TC

| Reference | Degradation performance | Operating conditions | Photocatalyst |
| --- | --- | --- | --- |
| ^2^ | Degradation efficiency of TC = 86.9% | TC concentration = 20 mg/L, catalyst dose = 50 mg/100 mL, light source = xenon lamp (300 W) and reaction time = 100 min | TiO_2_/BiOCl |
| ^3^ | TC degradation percentage = 79.4% | TC concentration = 20 mg/L, pH = 5.2, light source = xenon lamp (300 W), catalyst dose = 20 mg/60 mL and reaction time = 80 min | Meso-tetra (4-carboxyphenyl) porphyrin (TCPP)/Bi_12_O_17_Cl_2_ |
| ^4^ | TC degradation ratio = 85% | Catalyst dose = 20 mg/50 mL, TC concentration = 40 mg/L, light source = xenon lamp (300 W) and reaction time = 80 min | Fe_3_O_4_/BiOCl/BiOBr |
| ^5^ | TC degradation ratio = 80% | Catalyst dose = 50 mg/50 mL, TC concentration = 20 mg/L and reaction time = 100 min | Bi_12_O_17_Cl_2_/α-Bi_2_O_3_ |
| ^6^ | TC degradation percentage = 90.4% | TC concentration = 10 mg/L, catalyst dose = 0.1 g/100 mL, reaction time = 90 min and light source = xenon lamp (300 W) | BiOCl@Fe-MOF |
| ^7^ | TC degradation percentage = 90.3% | Reaction time = 80 min, light source = xenon lamp (300 W), TC concentration = 20 mg/L, pH =4 and catalyst dose = 40 mg/100 mL | Cu_2_O/BiOCl |
| ^8^ | TC degradation ratio = 91% | Reaction time = 60 min, TC concentration = 30 mg/L, catalyst dose = 30 mg/50 mL and light source = xenon lamp (1000 W) | Bi_4_O_7_/Cu-BiOCl |
| ^9^ | Removal efficiency of TC = 80% | TC concentration = 20 mg/L, catalyst dose = 20 mg/100 mL, light source = mercury lamp (300 W) and reaction time = 30 min | Carbon nanosheet/MnO_2_/BiOCl |
| This study | TC removal and COD mineralization ratios = 85.8% and 77.7%, respectively | TC concentration = 167 mg/L, COD = 1044 mg/L, catalyst dose = 150 mg/L, light source = solar light (30 W/m^2^), pH 4.7±0.5 and reaction time = 3 h | Biochar/Bi_12_O_17_Cl_2_ |

**References**

1. Stepanovich, A. Combinatorial development of porous semiconductor thin film photoelectrodes for solar water splitting by dealloying of binary and ternary alloys. Minsk, Belarus ( Weißrussland ) Bochum (2013).

2. Zou, X. *et al.* Enhanced visible-light photocatalytic degradation of tetracycline antibiotic by 0D/2D TiO2(B)/BiOCl Z-scheme heterojunction: Performance, reaction pathways, and mechanism investigation. *Appl. Surf. Sci.* **630**, 157532 (2023).

3. Wang, C., Yan, R., Cai, M., Liu, Y. & Li, S. A novel organic/inorganic S-scheme heterostructure of TCPP/Bi12O17Cl2 for boosting photodegradation of tetracycline hydrochloride: Kinetic, degradation mechanism, and toxic assessment. *Appl. Surf. Sci.* **610**, 155346 (2023).

4. Dang, J. *et al.* Fabrication of magnetically recyclable Fe3O4/BiOCl/BiOBr nanocomposite with Z-scheme heterojunction for high-efficiency photocatalytic degradation of tetracycline. *Mater. Sci. Semicond. Process.* **158**, 107371 (2023).

5. Chen, J., Zhong, J., Li, J. & Qiu, K. Boosted photocatalytic removal of tetracycline on S-scheme Bi12O17Cl2/α-Bi2O3 heterojunctions with rich oxygen vacancies. *Appl. Surf. Sci.* **563**, (2021).

6. Shi, Y. *et al.* Journal of Photochemistry & Photobiology , A : Chemistry In situ synthesis of donut-like Fe-doped-BiOCl @ Fe-MOF composites using for excellent performance photodegradation of dyes and tetracycline. *J. Photochem. Photobiol. A Chem.* **442**, 114704 (2023).

7. Yuan, X. *et al.* Preparation, characterization and photodegradation mechanism of 0D/2D Cu2O/BiOCl S-scheme heterojunction for efficient photodegradation of tetracycline. *Sep. Purif. Technol.* **291**, 120965 (2022).

8. Cui, Y., Zheng, J., Zhu, Z., Hu, C. & Liu, B. Preparation and application of Bi4O7/Cu-BiOCl heterojunction photocatalyst for photocatalytic degradation of tetracycline under visible light. *J. Mol. Struct.* **1274**, 134486 (2023).

9. Hong, X., Li, Y., Wang, X., Long, J. & Liang, B. Carbon nanosheet/MnO2/BiOCl ternary composite for degradation of organic pollutants. *J. Alloys Compd.* **891**, 162090 (2022).

1. © Corresponding author : Ahmed Tawfik ([prof.tawfik.nrc@gmail.com](mailto:prof.tawfik.nrc@gmail.com)) [↑](#footnote-ref-1)
